# Vaccine Immunity Against Pneumococcus in Children With Sickle Cell Disease: A Retrospective Single-center Study

**DOI:** 10.1097/INF.0000000000004947

**Published:** 2025-08-29

**Authors:** Clara Noble, Renato Gualtieri, Veneranda Mattiello, Laurent Cimasoni, Geraldine Blanchard-Rohner

**Affiliations:** From the * Faculty of Medicine; † Department of Pediatrics, Gynecology and Obstetrics, Research Platform of Pediatrics, Gynecology, and Obstetrics, University of Geneva, Geneva, Switzerland; ‡ Department Women-Mother-Child, Pediatrics Unit, Lausanne University Hospital and University of Lausanne, Lausanne, Switzerland; § Division of General Pediatrics, Department of Pediatrics, Gynecology and Obstetrics, Unit of Hematology and Oncology; ¶ Division of General Pediatrics, Department of Pediatrics, Gynecology and Obstetrics, Unit of Immunology, Vaccinology, and Rheumatology, Geneva University Hospitals and University of Geneva, Geneva, Switzerland.

**Keywords:** sickle cell, pneumococcus, vaccines, serology

## Abstract

**Background:**

Sickle cell disease (SCD) patients are at a higher risk of pneumococcal invasive diseases. Vaccination is the central strategy for protecting these children, along with penicillin prophylaxis. However, it is unclear how often these children should be revaccinated with pneumococcal vaccines. This retrospective study aimed to describe the pneumococcal vaccination status of children with SCD in a high-income country with access to vaccines, to see if the national vaccination guidelines are followed and effective at inducing good vaccine seroprotection. We also wanted to assess the longitudinal vaccine immunity and the effect of booster doses on vaccine seroprotection.

**Methods:**

Electronic medical records of 42 children with SCD diagnosed between 2009 and 2023 were retrospectively reviewed. Clinical demographic data and pneumococcal serologies were analyzed.

**Results:**

Of the 42 patients included in the study, 34 (81%) had available vaccine records. All of these patients had completed the age-appropriate vaccination schedule. Among them, 15 (44%) had received at least 1 booster dose, with a mean age of 3.47 years at the time of the booster. A Kaplan-Meier analysis revealed a significant decline in seroprotection after the age of 5 years following completion of the vaccination series.

**Conclusions:**

Our findings suggest that a booster vaccination may be necessary 5 years after the completion of the primary pneumococcal vaccination series. Further large-scale prospective studies are required to better define the optimal frequency of booster doses throughout life and to identify individual factors that contribute to the loss of serological protection.

Patients with sickle cell disease (SCD) are particularly at risk of infection due to poor splenic function, as the spleen undergoes infarction due to vaso-occlusive crises.^1,2^ Previous studies have shown that half of children with SCD develop functional asplenia by the age of 2 years.^3^ This impairs the spleen’s ability to respond to infections, to clear circulating antigens and to perform opsonization, thereby reducing the capacity of the immune system to neutralize encapsulated bacteria, especially pneumococcus.^4–6^

Pneumococcus are responsible for common and severe bacterial infections and are one of the leading causes of sepsis, bacterial pneumonia and meningitis.^7,8^ Children under 5 years and immunocompromised individuals are especially at risk of invasive pneumococcal diseases. Research indicates that among children under 5 years with SCD who died from infections, pneumococcus is the most common cause.^9^

Two pillars of pneumococcal infection prevention in SCD patients are antibiotic prophylaxis until 5 years and vaccination, both of which reduce morbidity and mortality.^10,11^ However, the rise of antibiotic-resistant pneumococcal strains highlights the urgency of a robust immunization program.^12,13^

In Switzerland, the Federal Commission for Vaccination recommends pneumococcal vaccination with conjugate vaccines for children under 5 years old, as well as for older children and adults with health conditions that increase their risk of invasive pneumococcal disease, such as SCD. In Switzerland, the transition from the 23-valent pneumococcal polysaccharide vaccine (PPV23) to pneumococcal conjugate vaccines (PCVs) for children began in the early 2000s, and PPV23 was formally no longer recommended in 2014.^14^ This shift was driven by the superior immunogenicity and effectiveness of PCVs in young children.^15^ Until 2025, the current pneumococcal vaccine recommendations for children include 3 doses of the pneumococcal conjugate vaccine PCV7 or PCV13 at 2, 4 and 12 months, with a catch-up vaccination depending on the age (Table 1).^16,17^ It is worth noting that, although the CDC recommends PPSV23 in addition to PCVs for at-risk children over 2 years to cover non-PCV13 IPD isolates,^18^ this is not implemented in Switzerland.

**TABLE 1.** Swiss vaccination recommendations for patients at risk of invasive pneumococcal diseases^16,17^

Age at the Start of Vaccination (Months of Age)	Total Number of Doses	Recommended Vaccination Schedule
2	3	2–4–12 months of age
3–5	3	Dose 1 and 2 at least 2 months apart, dose 3 at 12 months of age
6–11	3	Dose 1 and 2 at least 1 month apart, dose 3 at least 6 months after dose 2
12–23	2	Dose 1 and 2 at least 2 months apart
>24 months and adults	1	1 dose

No booster doses of pneumococcal vaccines are currently recommended.

The optimal immunization schedule to ensure long-term immunity in SCD patients, especially regarding the frequency of booster vaccinations with PCV, has not been identified yet. Swiss guidelines currently do not include booster shots for immunocompromised individuals, including SCD patients, due to a lack of data on their benefits.^19^

In our center, children with SCD undergo regular follow-up (every 1 or 2 years), which includes the measurement of pneumococcal antibodies. A booster vaccination is offered if the child does not meet the protection threshold. However, this follow-up is not universally available, particularly in resource-limited settings where SCD is most prevalent. Thus, understanding the appropriate timing for booster vaccinations is essential to provide the best care for all SCD patients.

To achieve this goal, it is crucial to determine how often SCD patients should be revaccinated with PCVs to maintain sufficient pneumococcal immunity throughout their life.

The aims of this study were to evaluate the pneumococcal vaccination status of children with SCD in a high-income country with access to vaccines, national immunization guidelines, and serological antibody testing. Additionally, the study aimed to assess longitudinal immunity over time to help determine the optimal timing for booster administration. Finally, it sought to provide observational data on the long-term effectiveness of pneumococcal booster vaccinations in maintaining protective immunity.

## METHODS

### Study Setting and Population

This retrospective observational study was conducted at Geneva University Hospitals in Switzerland. Children with SCD diagnosed between 2009 and 2023, less than 16 years of age at diagnosis, were eligible if their pneumococcal vaccine serology was available. The study was approved by the Geneva Regional Ethics Committee (CCER 2020-01537).

### Data Source and Collection

Demographic, vaccine and serology data were collected between September and October 2023 from the electronic medical records of each patient, using information from their routine appointments.

### Definition of Immunity and Vaccinal Status

All vaccine serology tests were analyzed in our vaccinology laboratory. Antibodies to *S. pneumoniae* were assessed for 3 representative serotypes (14, 19F and 23F) contained in the 13-valent conjugated vaccine (Prevenar13), using an enzyme-linked immunosorbent assay^20^ or a multiplex binding assay after 2019.^21,22^ These 3 pneumococcal serotypes were specifically and consistently analyzed over the study follow-up. Patients with serotype-specific immunoglobulin G (IgG) levels >0.30 mg/L in response to at least 2 of the 3 tested serotypes were considered “seroprotected.”^23^

The vaccination status was defined as complete according to the vaccine schedule defined in Table 1.

Regarding boosters, “not boosted” refers to individuals who had received at least the complete vaccination series, but no additional vaccine dose at the time of antibody measurement and “boosted” if they had. Regarding the antibody threshold for boosting, patients received booster doses of pneumococcal vaccine, based on clinical recommendations, serology results, and the doctor’s judgment. In clinical practice, the 0.3 mg/L cutoff was applied whenever possible; however, some boosters were also administered empirically.

### Statistical Analysis

Continuous data were presented when appropriate as median and interquartile ranges (IQRs) (for age) or geometric mean concentrations with the 95% CI (for antibody levels). Categorical data were presented as frequencies and percentages.

Seroprotection against Pneumococcus was also evaluated in a survival analysis, in which patients were censored when they had received a booster dose.

To investigate the role of the vaccine’s booster dose and the evolution of seroprotection over time, patients were divided into 2 groups based on whether they had received a booster dose in addition to the primary vaccination. The geometric means and confidence intervals of specific IgG against each serotype were then calculated starting from the age of 2 for each year of life.

The Kaplan-Meier (KM) curve depicts the natural evolution of seroprotection with age: in this analysis, the event was defined as the loss of seroprotection, and patients were censored now they received a booster vaccination.

Given the descriptive nature of our study, a sample size calculation was not performed; instead, all patients followed at our center who met the inclusion criteria were pragmatically included.

All analyses were conducted using Stata version 17 (2021; StataCorp) or RStudio (Posit Team, 2025, www.posit.co). All associated graphs were produced using Graph-Pad Prism version 6.04 for Windows (GraphPad Software, www.graphpad.com) or RStudio (Posit Team, 2025, www.posit.co).

## RESULTS

### Patient Characteristics

In total, 42 children with SCD, between 0 and 19 years of age, were followed between August 2007 and September 2023. Patient characteristics are reported in Table 2. The median age at the time of sampling was 10.69 (IQR: 5.54) years. A total of 24 of 42 (57%) patients were from Africa and 12/42 (29%) were from Europe. Twenty-six of 42 (62%) were female. Most of our patients, 32/42 (76%), had the HbSS genotype. Regarding clinical characteristics, 24/42 (56%) had received a transfusion, 32/42 (76%) benefited from hydroxyurea treatment, 8/42 (19%) experienced splenic sequestration and 5/42 (12%) had a splenectomy. None of the patients had undergone hematopoietic stem cell transplantation.

**TABLE 2.** Demographics Clinical Characteristics of Included Patients

	Total (N = 42)	Available Vaccine Data (N = 34)	Vaccine Data Absent (N = 8)	*P*-value
Female, n (%)	26 (62)	18 (53)	8 (100)	0.8901
Age, years, median (IQR)	10.69 (5.54)	9.73 (6.36)	11.05 (3.29)	
Hemoglobin type				
HbSS, n (%)	32 (76)	27 (79)	5 (63)	0.2095
HbSC, n (%)	10 (24)	7 (21)	3 (27)	
Nationality by continent				
Africa, n (%)	24 (57)	21 (62)	3 (27)	0.3501
North America, n (%)	3 (7)	2 (6)	1 (13)	
South America, n (%)	3 (7)	3 (7)	0	
Asia, n (%)	0	0	0	
Europe, n (%)	12 (29)	8 (24)	4 (50)	
Transfusions received	24 (57)	19 (56)	5 (63)	0.7816
Hydroxyurea treatment	32 (76)	27 (79)	5 (63)	1.0000
Experienced splenic sequestration	8 (19)	8 (24)	0	1.0000
Splenectomy	5 (12)	5 (15)	0	0.7536

### Vaccine Status

**FIGURE 1:**
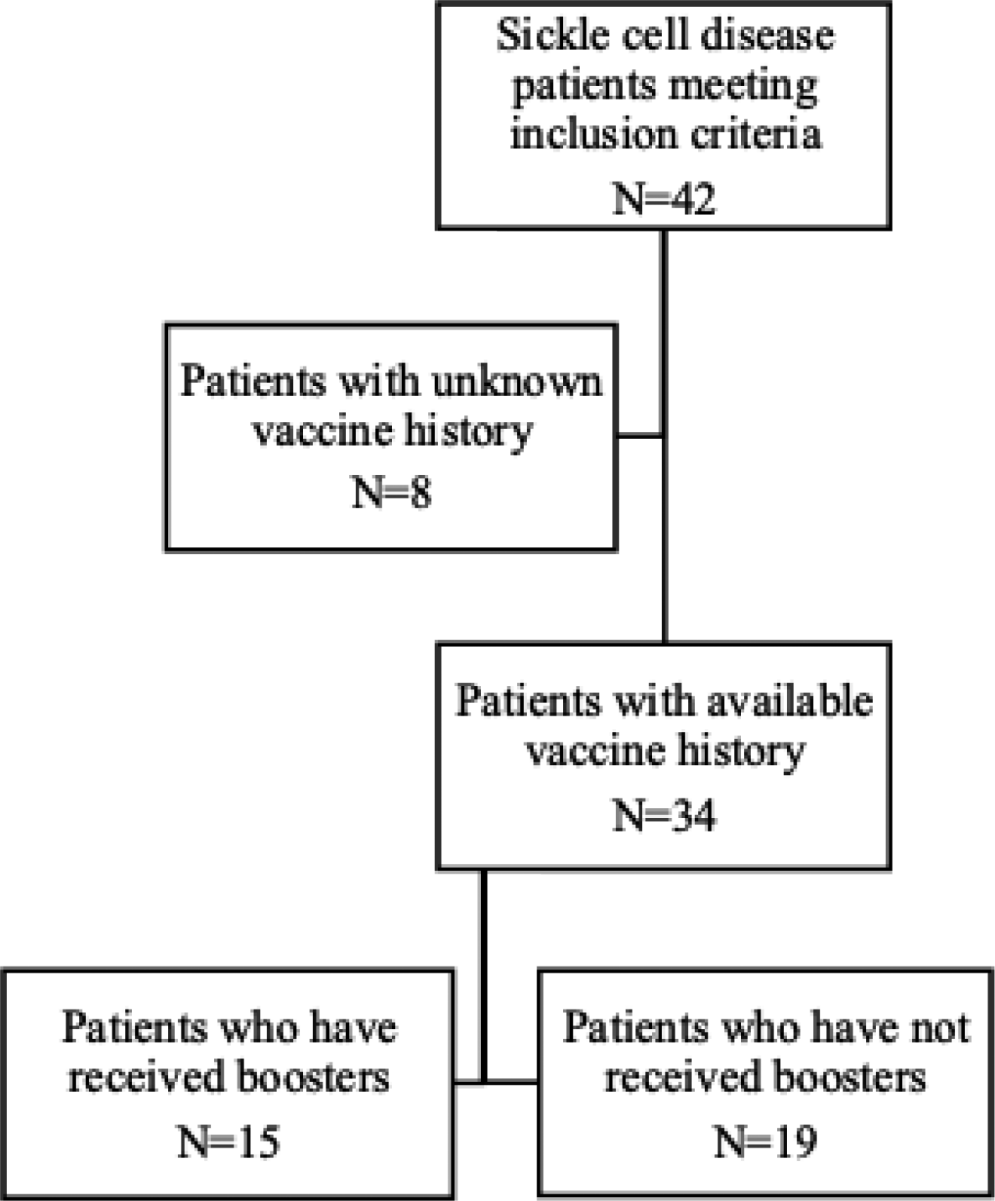
Study population flowchart.

Of the 34/42 (81%) patients who had available vaccination records, all were fully vaccinated for their age. Among these patients, 15/34 (44%) had received at least 1 booster dose, at median age of 3.47 (IQR: 4.30) years old (Table 3 and Fig. 1).

**TABLE 3.** Vaccine and Serology Characteristics of Included Patients

Available Vaccine History, n (%)	34/42 (81)
Vaccines up-to-date for age*	34/34 (100)
Vaccine type for base vaccination*	
PCV13, n (%)	30/34 (88)
PPV23, n (%)	4/34 (12)
Combination PCV13 & PCV7, n (%)	1/34 (3)
Age when boosters were received*†	
Before age 5, n (%)	5/34 (15)
After age 5, n (%)	4/34 (12)
Before and after age 5, n (%)	6/34 (18)
No Booster, n (%)	19/34 (56)
Age when first boosted, years, median (IQR)*†	3.47 (4.30)
Vaccine type for booster vaccination*†	
PCV13, n (%)	10/15 (67)
PPV23, n (%)	1/15 (7)
Combination PCV13 & PPV23, n (%)	4/15 (27)
Patients who had multiple serology during the study follow-up, n (%)	23/42 (55)
Mean number of serology, n (IQR)	3 (11)

* For patients with available vaccine history, defined as “complete primary vaccination according to Table 3.”

† Patients who had received the recommended number of doses for age, and at least 1 additional vaccine dose.

### Booster Administration and Vaccine Serology Monitoring

In Supplemental Digital Content 1, https://links.lww.com/INF/G329, the longitudinal seroprotection levels of patients who received boosters are represented.

Antibody response to booster vaccinations was typically evaluated 4–6 weeks postadministration, or at the next follow-up visit for practicality. The response was robust in all children tested within this 4–6-week period.

### Types of Pneumococcal Vaccine Used

In our sample, only 4 patients had received their primary vaccination with the PPV23, and 2 had received the PPV23 as boosters. Otherwise, the PCVs PCV13 or PCV7 were used. Given this limited number of cases, it was not possible to assess for a “PPV23 booster effect.”

### Longitudinal Pneumococcal Vaccine Immunity

During the follow-up, the 42 included patients had on average 3 (IQR: 11) pneumococcal serology, on average every 1 or 2 years. They were on average 6 years old at the time of their first pneumococcal serology. Among patients with available vaccine records, 10/34 (29%) patients had no immunity against pneumococcus, despite having a documented up-to-date pneumococcal vaccination for age.

**FIGURE 2:**
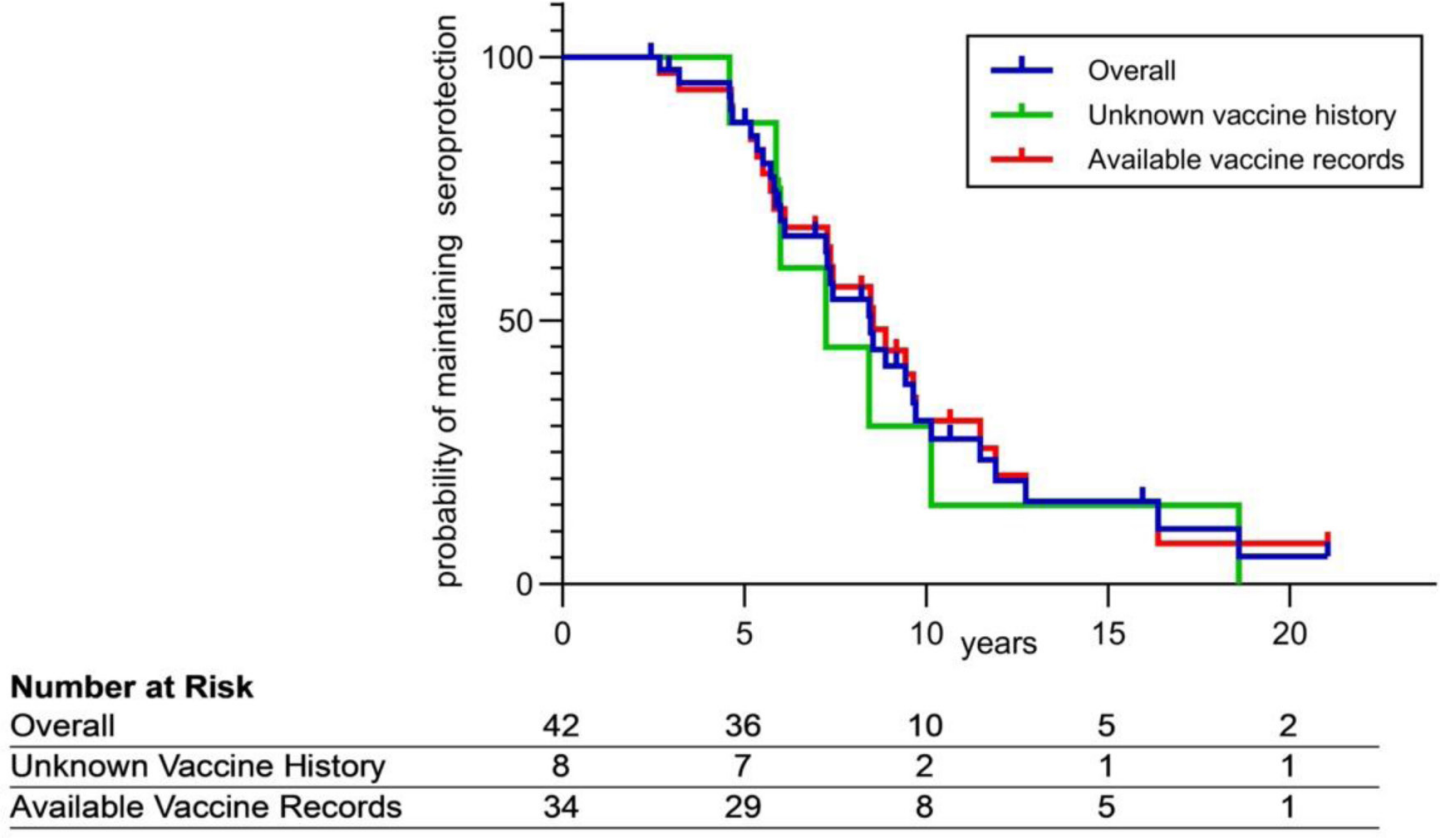
Kaplan-Meier curve estimating the probability of maintaining seroprotection over time. Kaplan-Meier curve shows the probability of maintaining seroprotection over time in a group of 42 study subjects before booster vaccination. The X-axis shows the number of individuals with a probability of maintaining seroprotection at specific timepoints (0, 5, 10, 15 and 20 years of age). The Y-axis represents the estimated probability of patients to maintain seroprotection at each of these timepoints, with 100% indicating complete seroprotection. Loss of seroprotection was defined as not having serotype-specific immunoglobulin G (IgG) levels >0.30 mg/L for at least 2 of the 3 tested serotypes. The entire population is described in blue, the subgroup with available vaccination records in green and the subgroup without available vaccination records in red.

The KM analysis shows that, starting from a high level of seroprotection, there is a dramatic loss of seroprotection beginning at the age of 5 years. By 10 years, most patients had lost their seroprotection (Fig. 2). This observation is applicable to the whole population, as the KM curve for the entire population overlaps with that of the subgroup with vaccination records and the subgroup without vaccination records.

**FIGURE 3:**
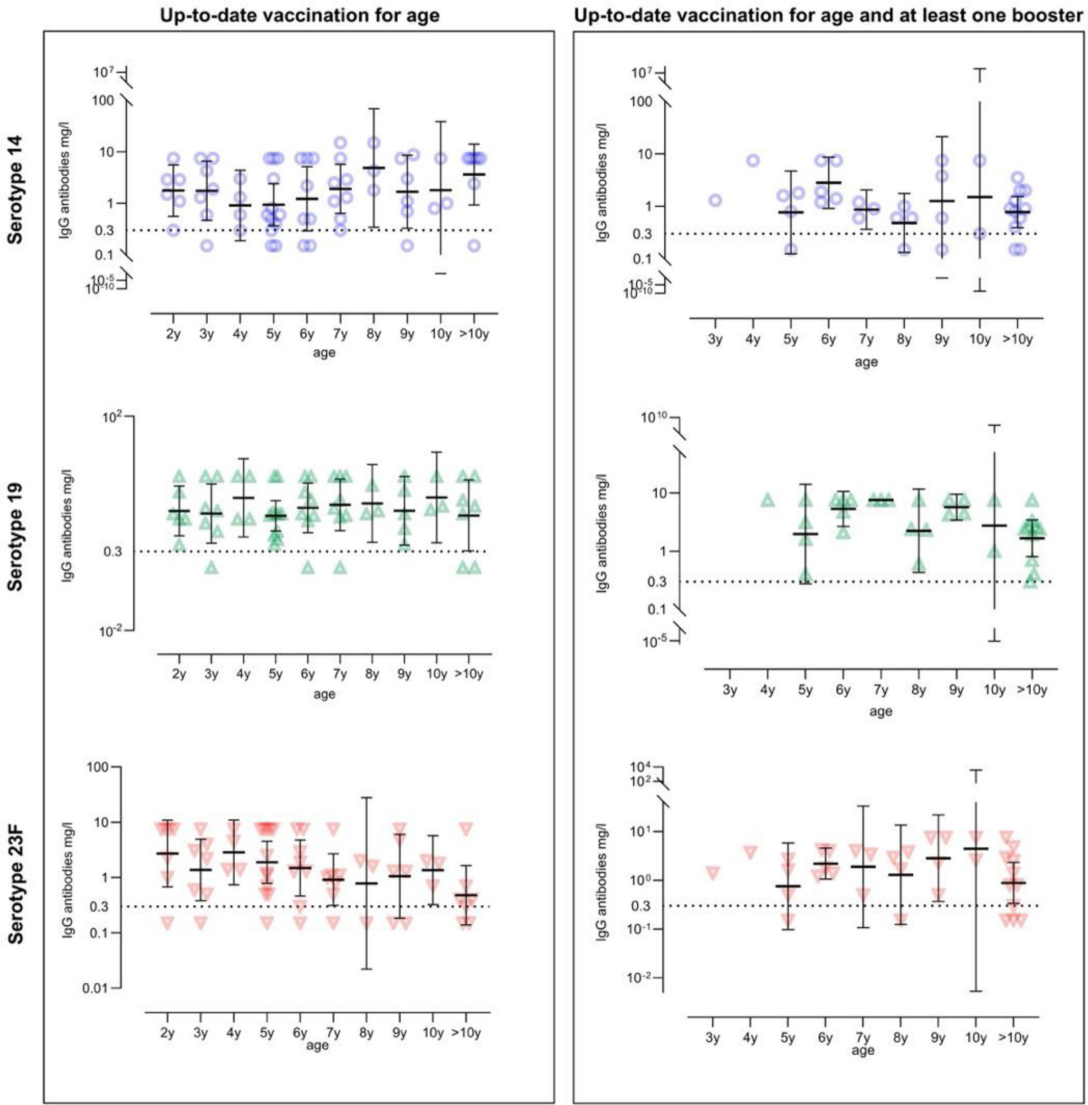
Pneumococcal IgG levels of the 34 patients with available vaccination records during the study follow-up, according to booster vaccination status and pneumococcal serotype. Each symbol represents an individual patient’s serological value. The dotted line marks the immunity threshold of 0.3 mg/L correlating with seroprotection,^20^ and error bars indicate the 95% confidence intervals.

It should be noted that the KM curve and longitudinal pneumococcal IgG levels are not directly comparable: the KM provides a longitudinal estimation of the duration of seroprotection in the absence of boosters, whereas data summarized in Figure 3 and Supplemental Digital Content 2, https://links.lww.com/INF/G329, represents a cross-sectional snapshot of seroprotection prevalence at each age group, irrespective of prior serological status.

When analyzed according to age, seroprotection levels for individual serotypes were, on average, lower in children who had not yet received a booster vaccination, compared to those who had, although these observations are descriptive rather than statistically tested due to the limited sample size (Fig. 3 and Supplemental Digital Content 2, https://links.lww.com/INF/G329). When looking at individual serotypes, there seems to be a faster fall of immunity for serotype 23F, compared with serotypes 14 and 19. Comparison of aggregate IgG levels before and after 5 years of age did not provide a statistically significant difference (Supplemental Digital Content 3, https://links.lww.com/INF/G329). When divided into 3 age groups (<5, 5–10 and >10 years), similar patterns were observed, with no significant difference in pneumococcal seroprotection between boosted and nonboosted individuals across age groups (Supplemental Digital Content 4, https://links.lww.com/INF/G329).

Our analysis reveals that none of the serotypes show a statistically significant difference between before and after 5 years of age (Supplemental Digital Content 3, https://links.lww.com/INF/G329).

## DISCUSSION

All patients with available vaccination history had an up-to-date pneumococcal vaccination for age. However, close to 20% of our patients had no vaccination history available (vaccination booklet), which is concerning. Indeed, vaccination history is crucial for defining the best vaccination schedule for each patient. The lack of data could be due to several factors, including the absence of standardized vaccine records between countries, loss, or misplacement of records by families, incomplete recording due to a follow-up by several doctors/hospitals without national immunization records. These challenges are well known and could be improved, for example by introducing an electronic vaccination records accessible to the families and doctors at any time.^24–26^ It is important to note that Switzerland had an electronic vaccine registry available until the COVID-19 pandemic, but it was deactivated due to concerns about a potential data breach. Despite this, the electronic registry was never mandatory for all patients, and vaccination cards have continued to serve as the primary method for recording immunization history, both historically and currently in Switzerland. However, there is a need to consider implementing a secure electronic national vaccination registry to track vaccinations for all patients, particularly those under the care of multiple healthcare providers.

In our study, one-third of the patients lacked pneumococcal vaccine-induced immunity, consistent with previous research showing that approximately a third of individuals with SCD have suboptimal levels of seroprotection.^24–27^ The marked decline in immunity observed after 5 years suggests that a booster dose may be warranted at that time. However, this study did not enable us to identify individual factors associated with the loss of serological protection. Regarding serotype-specific immune response to pneumococcal vaccine, other studies have reported a variation in response, although the mechanism remains unclear.^27^ One possibility is that B-cell responses may vary between pneumococcal serotypes, with B cells specific to certain serotypes exhibiting different characteristics. For example, serotype 23F has been reported to be less immunogenic than serotypes 14 and 19,^28^ which is consistent with our findings. We observed that there was no significant difference in maintenance of pneumococcal immunity over time, between patients who had received a booster vaccination and those who had not. However, there was a tendency towards a higher concentration of pneumococcal IgG levels among the children who had received a booster dose of pneumococcal vaccine.

Our study has several limitations. First, its retrospective nature hindered the collection of all data points of interest, particularly the number and frequency of serology data and detailed vaccination history. Additionally, clinical events such as otitis, invasive pneumococcal disease, and pneumonia could not be collected and, therefore, could not be correlated with serologic values. It should also be noted that we did not stratify analysis according to the type of vaccination received, despite PCV13 being the only vaccine recommended after 2014, while our data collection started in 2007. This could be a particular point of interest for future research, as a recent randomized controlled trial in adults by Melica et al^28^ underlined the improved response when a PCV13/PPSV23 regimen was used. Notably, in their study, both the PCV13/PPSV23 and PPSV23 groups maintained seroprotection at the 24-month follow-up. In contrast, our data show a decline in seroprotection across the entire study population, underscoring the need to explore the vaccine regimen further in the pediatric population. The absence of a universally accepted cutoff for pneumococcal immunity also limits the generalizability of this analysis; however, the cutoff of 0.3 mg/L is widely used.^23^ Moreover, the small sample size limits the study to providing primarily descriptive data, without the ability to draw statistically significant conclusions. It is also important to note that the heterogeneity of our sample (for example regarding nationality, hemoglobin type, age, pattern of vaccination) impedes on the interpretation and generalizability of our results.

Overall, this study demonstrated that most SCD patients maintained sufficient serological protection against pneumococcus during the first 5 years of life. However, after this time, the risk of losing pneumococcal vaccine immunity increased rapidly. Future longitudinal, prospective larger studies should be performed to better describe at which frequency booster vaccination against pneumococcus should be given to SCD patients, and to help identify potential individual characteristics associated with loss of pneumococcal immunity.

## ACKNOWLEDGMENTS

*The authors express their gratitude to the Pediatric, Gynecology, and Obstetrics research platform of the Geneva University Hospital for their support and collaboration, and to Nicolas Silvestrini for his assistance with statistical analysis.*

## Supplementary Material


