# Supplementary material for: Vaccine Immunity Against Pneumococcus in Children With Sickle Cell Disease: A Retrospective Single-center Study
Source: Pediatr Infect Dis J. 2025 Aug 29;45(1):68–73. doi: 10.1097/INF.0000000000004947 (PMC12688463; doi:10.1097/INF.0000000000004947)
Supplement: Supplementary file 1 [file inf-45-68-s001.pdf]

SUPPLEMENTAL DIGITAL CONTENT 1. Longitudinal seroprotection among patients with multiple serologies who had received at least one booster.

Legend: Each color represents a different patient, with each dot corresponding to a serological value. The dotted vertical lines indicate the timing of each patient's booster. The dotted horizontal line marks the immunity threshold of 0.3 mg/l correlating with seroprotection (20)

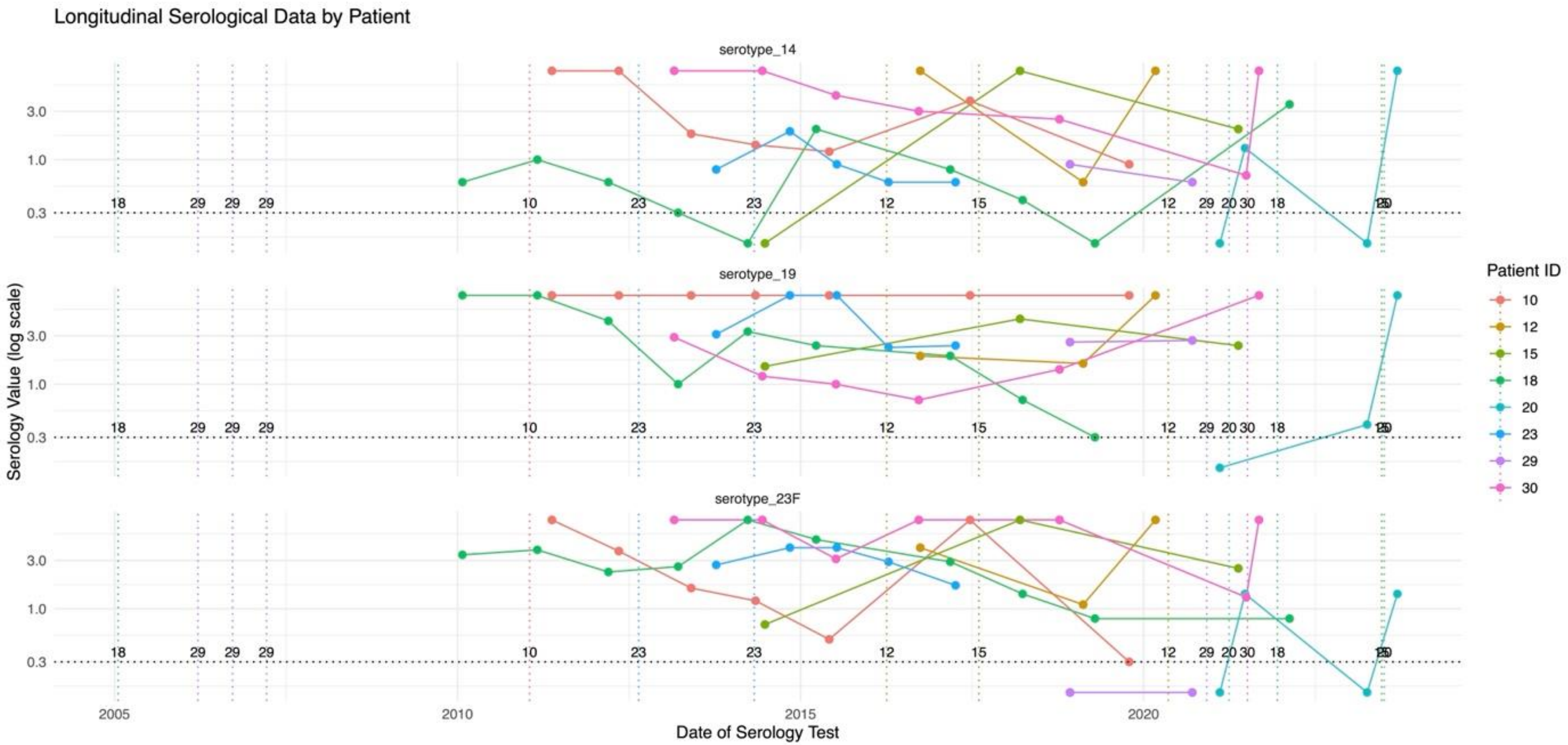

SUPPLEMENTAL DIGITAL CONTENT 2. Pneumococcal serotype-specific-IgG levels during the study follow-up of the 34 patients with available vaccination records during the study follow-up, according to booster vaccination status and pneumococcal serotype.

**Age Group**	**N**	**N Serotype 14**	**Serotype 14 Median (IQR)**	**N Serotype 19F**	**Serotype 19F Median (IQR)**	**N Serotype 23F**	**Serotype 23F Median (IQR)**
Up-to-date vaccination for age without booster*							
1	1	1	7.5 (0)	1	2.9 (0)	1	7.5 (0)
2	6	6	2.2 (1.7)	6	1.35 (2.025)	6	4.9 (6.175)
3	11	11	3.2 (6.1)	11	2 (4.3)	11	2.7 (3.05)
4	4	4	0.95 (1.2)	4	4.35 (6.3)	4	2.95 (3.85)
5	13	13	0.6 (2.6)	12	1.45 (0.95)	13	1.2 (6.8)
6	11	11	0.5 (7.35)	10	2.15 (5.4)	11	1.5 (4.4)
7	10	10	1.75 (2.15)	10	2.3 (6.175)	10	1.05 (1.5)
8	4	4	2.05 (3.8)	4	3.4 (4.05)	4	1 (1.3625)
9	6	6	2.05 (5.575)	5	1.5 (3.3)	6	1.3 (3.4875)
10	5	5	1.8 (1.4)	3	2.1 (2.85)	5	1.5 (0.7)
>10	8	8	0.15 (0)	8	0.15 (0)	8	0.4 (0)
Up-to-date vaccination for age and at least one booster							
3	1	1	1.3 (0)	0	NA (NA)	1	1.4 (0)
4	1	1	7.5 (0)	1	7.5 (0)	1	3.7 (0)
5	4	4	1.2 (1.0125)	4	2.35 (2.9)	4	1.05 (1.4625)
6	5	5	1.9 (6.1)	5	7.4 (2.8)	5	1.8 (2.6)
7	3	3	0.9 (0.3)	3	7.5 (0)	3	3.4 (1.75)
8	4	4	0.6 (0.2125)	4	2.35 (1.8)	4	2.3 (1.8125)
9	4	4	2.2 (4.2375)	4	5.95 (3.15)	4	4.9 (5.65)
10	2	2	3.9 (3.6)	2	4.25 (3.25)	2	5.05 (2.45)
>10	11	11	0.525 (0.375)	11	5.4 (2.1)	11	3.9 (3.6)

*Up to date vaccination for age is defined as "complete primary vaccination according to table 3".

**Note that there is not age group 1 and 2 for the up-to-date vaccination for age and at least one booster.

SUPPLEMENTAL DIGITAL CONTENT 3. Aggregated IgG levels by pneumococcal serotype, comparing patients who had received a booster vaccination before 5 years of age versus those who had received it after 5 years of age.

Legend : Data represents group-level seroprotection measures compiled from all available patient samples. This comparison evaluates whether the timing of the booster dose before or after age 5 influences the magnitude of serotype-specific immune responses. It was performed using the T-test. No significant differences in IgG levels were observed between the two groups, suggesting that booster timing relative to the age of 5, does not substantially impact seroprotection.

**Serotype**	**Booster timing**	**Number of serologies (n)**	**Mean Level**	**Standard Deviation**	**Standard Error (SE)**	**CI (Lower)**	**CI (Upper)**	**p-value**	**t-statistic**	**CI for Difference (Lower)**	**CI for Difference (Upper)**
**Serotype 14**	Before 5 Years	28	3.09	2.78	0.525	2.06	4.12	0.946	0.0677	-1.31	1.41
	After 5 Years	67	3.13	3.55	0.434	2.28	3.98				
**Serotype 19**	Before 5 Years	26	2.99	2.68	0.526	1.95	4.02	0.151	1.46	-0.351	2.21
	After 5 Years	62	3.92	2.85	0.361	3.21	4.63				
**Serotype 23F**	Before 5 Years	28	3.49	2.81	0.531	2.45	4.54	0.151	-1.46	-2.14	0.339
	After 5 Years	67	2.59	2.56	0.313	1.98	3.21				

SUPPLEMENTAL DIGITAL CONTENT 4. Comparison of proportion of participants who are not seroprotected at the moment of serology according to booster status across age groups

Legend : The table presents the proportion of individuals classified as not protected against pneumococcus, stratified by booster vaccination status at the time of serology across three age groups (>3 years). Values are shown as the number and percentage of unprotected individuals within each booster group. Statistical comparison between « boosted » and « non-boosted » groups for each age category was performed using Fisher's exact test. This reveals no statistically significant differences between groups.

**Age group at the moment of serology (years)**	**Number of serologies, n**	**Booster Status at the moment of serology, n (%)**		**Not protected, n (%)**	**p-value (Fisher's exact test)**
3–5	9	With booster	3 (33)	1 (33)	1.0000
		Without booster	6 (66)	2 (33)	
6–10	24	With booster	4 (17)	1 (25)	1.0000
		Without booster	20 (83)	4 (20)	
>10	81	With booster	28 (35)	1 (4)	0.6544
		Without booster	53 (65)	4 (8)	
